# Dasabuvir alleviates 5-fluorouracil-induced intestinal injury through anti-senescence and anti-inflammatory

**DOI:** 10.1038/s41598-024-66771-x

**Published:** 2024-07-08

**Authors:** Siyue He, Zhiwei Wang, Jing Xia, Huijie Jia, Qianlong Dai, Cui Chen, Fei He, Xiaobo Wang, Min Zhou

**Affiliations:** 1 School of Basic Medicine, Dali University, Dali, 671000 Yunnan China; 2 Department of Biotherapy, Cancer Center and State Key Laboratory of Biotherapy, West China Hospital, Sichuan University, Chengdu, 610000 Sichuan China; 3 Qujing Medical College, Qujing, 655011 Yunnan China

**Keywords:** Cell biology, Molecular biology, Gastroenterology

## Abstract

5-Fluorouracil (5-Fu) is a basic drug that is used to treat colorectal cancer. Patients who receive 5-Fu chemotherapy often experience side effects that affect the digestive system, such as intestinal injury and diarrhoea, which significantly affect patient compliance with anticancer treatment and quality of life. Therefore, identifying approaches to treat or prevent these side effects is urgent. Dasabuvir (DSV) is a hepatitis C virus inhibitor, but its impact on 5-Fu-induced intestinal injury remains unknown. Our study investigated the effects of DSV on 5-Fu-induced intestinal injury in HUVECs, HIECs and male BALB/c mice. We found that 5-Fu caused intestinal damage by inducing senescence, increasing inflammatory factor expression, and generating oxidative stress. Compared with 5-Fu treatment alone, DSV inhibited senescence by reducing senescence-β-galactosidase (SA-β-gal) activity, the senescence-associated secretory phenotype (SASP, including IL-1, IL-6, and TNF-α) and senescence marker expression levels (p16, p21, and p53). Moreover, the anti-senescence effect of DSV was achieved by inhibiting the mTOR signaling pathway. DSV increased antioxidant enzyme levels and alleviated intestinal tissue injury in mice. In addition, DSV suppressed the 5-Fu-induced increase the diarrhoea scores and ameliorated the weight loss, food intake and water intake of the mice. Overall, this study indicated that DSV could be used to treat chemotherapy-induced intestinal damage.

## Introduction

5-Fluorouracil (5-Fu) is an important tumor treatment that is commonly used in the clinic, but its side effects seriously affect its efficacy and patient prognosis^1^. 5-Fu can cause gastrointestinal reactions, cardiovascular toxicity, bone marrow suppression and other side effects^2–4^. The incidence of mucositis caused by chemotherapy can reach 40%^5^. Common chemotherapy-induced gastrointestinal symptoms include nausea, vomiting, diarrhoea, and loss of appetite^6^. When these symptoms occur, patients are forced to reduce the chemotherapy dose or even discontinue treatment, resulting in increased mortality^7^. Therefore, the identification of drugs to repair intestinal injury and relieve intestinal mucositis is vital.

The pathogenesis of the side effects of 5-Fu-induced intestinal mucositis is not fully understood. 5-Fu acts as an anticancer agent by damaging DNA, resulting in the death of both cancer cells and normal cells^8^. Sonis et al. proposed that exposure of the gastrointestinal mucosa to chemotherapy agents leads to oxidative stress and the generation of reactive oxygen species, which in turn damage the intestinal barrier and tissues. Consequently, the activation of NF-κB triggers the release of proinflammatory cytokines (IL-1β, IL-6, and TNF-α), further exacerbating damage to intestinal tissues^9^. Currently, studies have indicated that intestinal mucositis can be prevented or alleviated through the use of anti-inflammatory drugs^10,11^. Therefore, the search for effective anti-inflammatory drugs holds promise for the management of this adverse reaction.

Senescence occurs when cells are exposed to radiation, chemotherapy, and oxidative stress, which damage the intestinal epithelium^12^. Radiation and chemotherapy treatments can cause cell senescence, and this condition is known as treatment-induced senescence (TIS)^13^. The persistence of cells with TIS has been shown to cause local and systemic inflammation^14^. Stress-induced senescence is a marker of anticancer drug-induced cardiovascular cytotoxicity. Studies have shown that the cardiovascular toxicity of fluorouracil is mediated by endothelial cell senescence, and such side effects could be antagonized by ameliorating endothelial cell senescence^15^. Depleting cells with adriamycin-induced TIS in mice alleviated side effects, including myelosuppression, cardiac dysfunction, and fatigue, and ameliorated the SASP^16^. Therefore, we hypothesized that the induction of intestinal senescence may be one of the causes of the side effects of 5-Fu. Therefore, drugs that suppress senescence may be useful for treating the side effects of chemotherapy.

mTOR is an evolutionarily conserved atypical serine/threonine protein kinase that is essential for regulating cell growth and metabolism. mTOR mainly includes mTORC1 and mTORC2, among which mtorc1 is sensitive to rapamycin and can regulate senescence through protein synthesis, autophagy, the SASP, etc^17^. Studies have shown that the inhibition of mTOR can prolong mammalian life and prevent aging-related diseases. Inhibition of mTOR has been shown to alleviate the doxorubicin- or hydrogen peroxide-induced senescence of human umbilical cord mesenchymal stem cells^18^. The activation of mTOR signalling can promote the onset of acute ulcerative colitis^19^.

Dasabuvir (DSV), which is an antiviral drug, is used to treat hepatitis C infection^20^. This study first found that DSV alleviates 5-Fu-induced senescence, intestinal damage, inflammation and oxidative stress in HUVECs, HIECs and BALB/C mice and alleviates senescence by inhibiting the expression of the mTOR signalling pathway.

## Results

### DSV alleviates 5-Fu-induced endothelial cell and intestinal epithelial cell senescence

**Figure 1:**
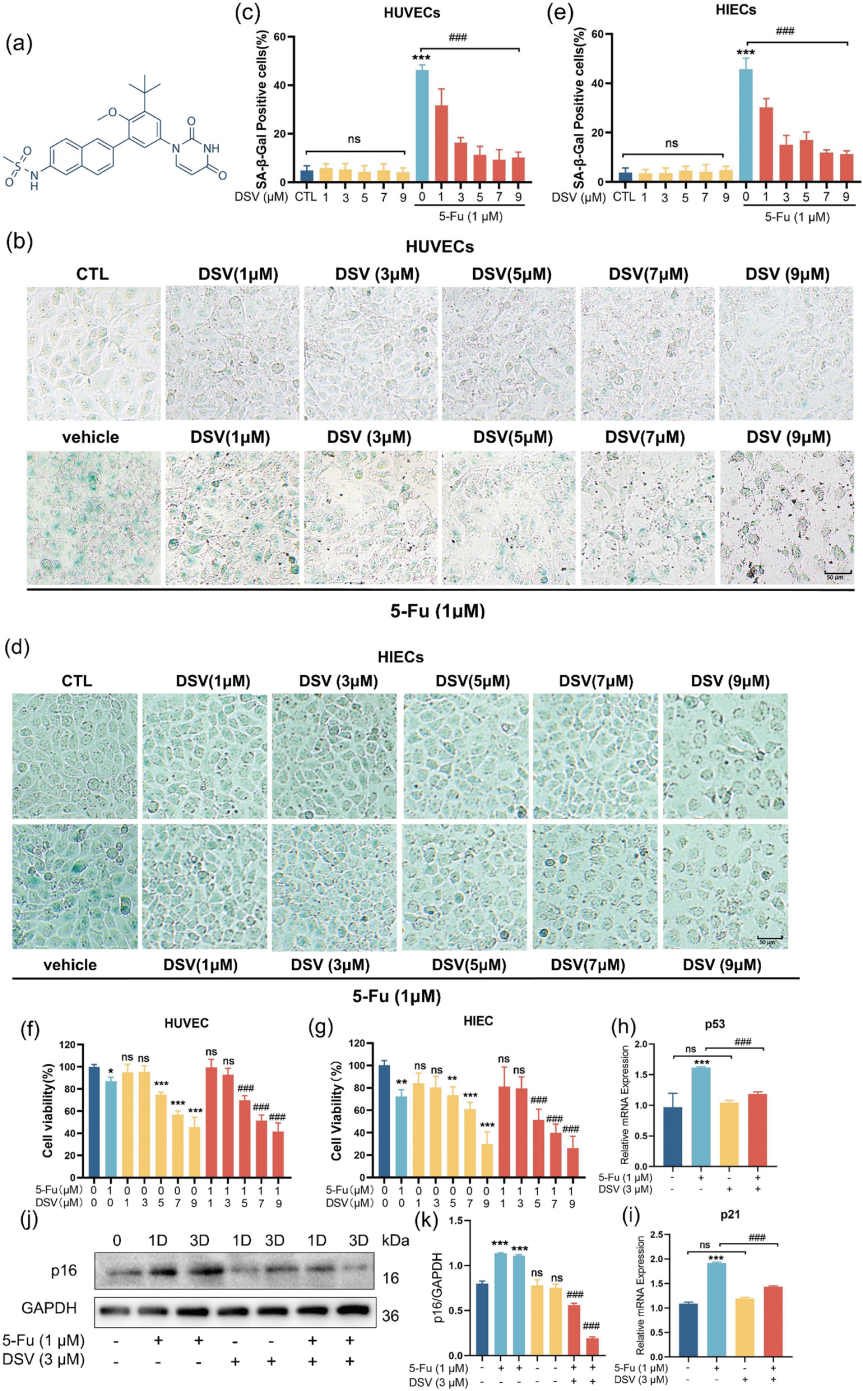
DSV inhibits senescence induced by 5-Fu in HUVECs and HIECs. (**a**) Structure of DSV. (**b,c**) Representative images and percentage of SA-β-gal-positive HUVECs cultured with 5-Fu, DSV, and DSV combined with 5-Fu (magnification ×50, scale bar: 50 µm, n = 3). (**d,e**) Representative images and percentages of SA-β-gal-positive HIECs cultured with 5-Fu, DSV, and DSV combined with 5-Fu (magnification ×50, scale bar: 50 µm, n = 3). (**f,g**) Cell viability of HUVECs and HIECs treated with 5-Fu (1 μM) plus different concentrations of DSV (n = 5). (**h,i**) p53 and p21 mRNA expression in HIECs cultured with 3 μM DSV or 1 μM 5-Fu and cocultured for 3 days (n = 3). (**j,k**) 1 day or 3 days after treatment with 3 μM DSV, 1 μM 5-Fu and coculture, HIECs were collected. Western blots showing expression levels of p16 (n = 3). The samples were derived from the same experiment, and the blots were processed in parallel. Original blots are presented in Supplementary Figs. S1 and S2. *p < 0.05, **p < 0.01, ***p < 0.001, compared with the control group; ^#^*p* < 0.05, ^##^*p* < 0.01, ^###^*p* < 0.001, compared with the 5-Fu group.

HUVECs are often used as a model to induce premature cellular senescence. Our published article reported that 5-Fu induced senescence in the highest percentage of cells at a concentration of 1 μM^21,22^. Therefore, 1 μM 5-Fu was used to establish a senescence model in HUVECs and HIECs. To study the effect of DSV on 5-Fu-induced senescence, HUVECs were incubated with different concentrations of DSV combined with 1 μM 5-Fu for 6 days. Senescence was assessed by determining the percentage of SA-β-gal-positive cells. While 5-Fu increased the percentage of SA-β-gal-positive cells, 3 to 7 µM DSV notably reduced the percentage of SA-β-gal-positive cells, indicating that DSV can alleviate endothelial cell senescence (Fig. 1a–c). We hypothesized that the intestinal side effects in patients who receive chemotherapy are related to the senescence-inducing effects of the chemotherapeutic agents. To test this hypothesis, we performed preliminary experiments using HIECs. 5-Fu induced the senescence of HIECs, while DSV reversed this increase in the proportion of SA-β-gal-positive cells (Fig. 1d, e). Compared with those in the control group, cells that were treated with DSV (5 µM or higher) exhibited a flattened morphology and less distinct cell outlines. However, after treatment with 3 µM DSV, the cell morphology appeared normal, with a low percentage of ageing cells. In addition, treatment with DSV alone did not induce HUVEC or HIEC senescence. Subsequently, we investigated the impact of coculture with drugs on cell viability. As shown in Fig. 1f, g, our findings revealed that there was no statistically significant effect on cell viability When DSV was used at concentrations ≤ 3 µM, whether DSV given alone or in combination with 5-Fu. DSV (3 μM) was selected for subsequent experiments. To further investigate the anti-senescence effect of DSV in HIECs, we examined the protein and gene expression of the senescence-related markers p16, p21 and p53. 5-Fu upregulated p16 protein expression and p53 and p21 mRNA expression in HIECs. Cotreatment with DSV downregulated the p16 protein expression and the p53 and p21 mRNA expression. Moreover, DSV alone did not affect these indicators (Fig. 1h–k). Hence, these results indicated that DSV alleviates 5-Fu-induced endothelial cell and intestinal epithelial cell senescence.

### DSV attenuates intestinal senescence in 5-Fu-treated mice

**Figure 2:**
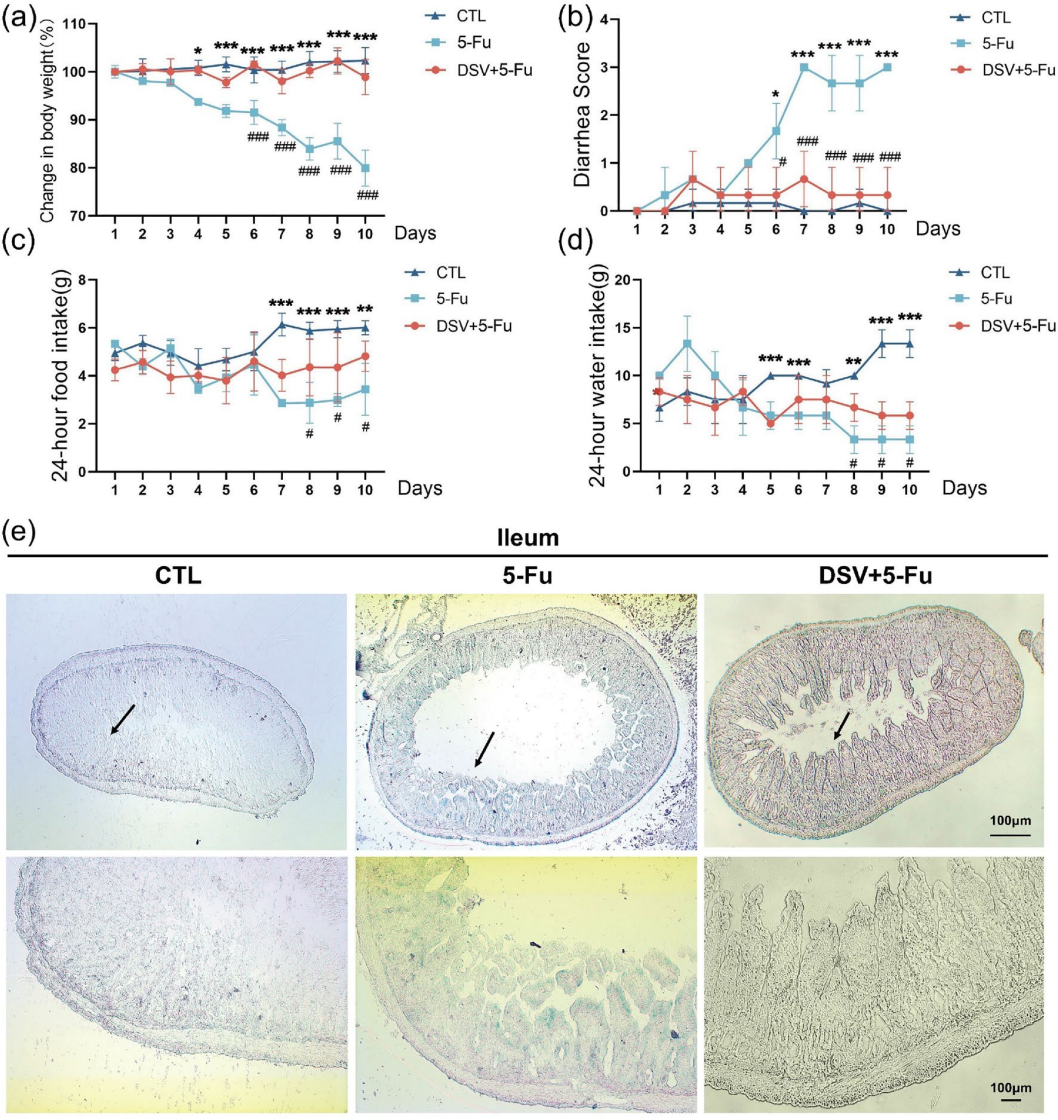

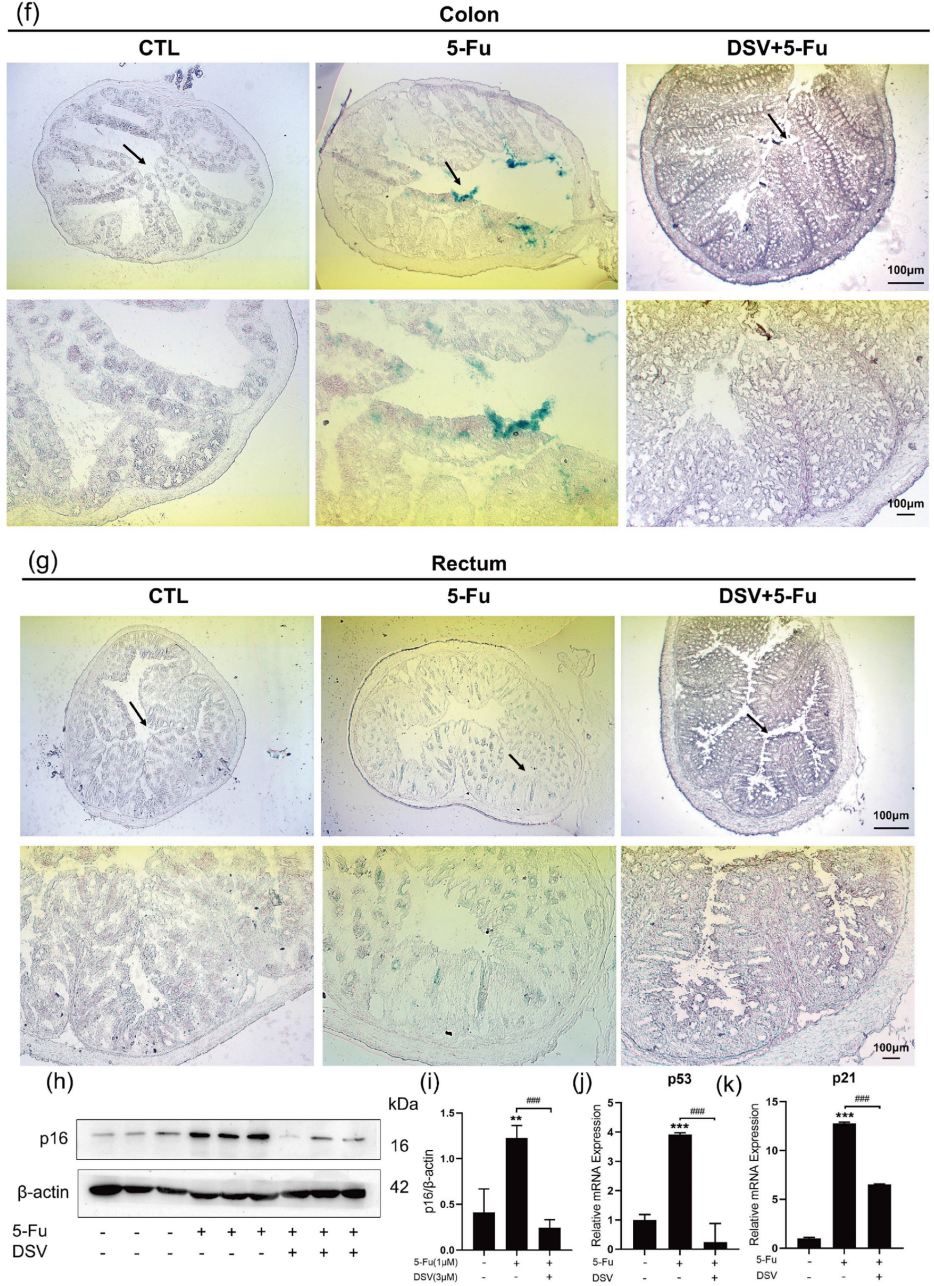
DSV inhibits intestinal senescence induced by 5-Fu. (**a–d**) Daily changes in body weight, diarrhoea score, food intake, and water intake in each group. Statistics were determined by Two-way ANOVA. The sample size is n = 6. Intestinal tissue (from the ileum, colon and rectum) was collected after administration. (**e–g**) Representative images of SA-β-gal-stained intestinal tissue sections. (Scale bars, 100 μm; upper images, ×40  magnification; lower images, ×100  magnification; n = 3). (**h,i**) Western blots and expression levels of p16. The samples were derived from the same experiment, and the blots were processed in parallel (n = 3). Original blots are presented in Supplementary Figs. S3 and S4. (**j,k**) mRNA expression levels of p53 and p21(n = 3). *p < 0.05, **p < 0.01, ***p < 0.001, compared with the control group; ^#^*p* < 0.05, ^##^*p* < 0.01, ^###^*p* < 0.001, compared with the indicated group.

Next, we further investigated the anti-senescence effects of DSV by establishing a diarrhoea mouse model. During treatment, several clinical parameters and signs were recorded. Compared with those in the control group, the mice that were treated with 5-Fu lost significantly more weight, while DSV prevented weight loss. (Fig. 2a) (body weight of each group before sacrifice: control group: 30.88 g ± 1.043, 5-Fu group: 23.30 g ± 0.952, DSV group:26.20 g ± 0.671). Starting on the second day, the mice in the 5-Fu group began to develop diarrhoea. From Days 6 to 10, the diarrhoea score of the 5-Fu group decreased in response to DSV treatment (Fig. 2b). DSV ameliorated the 5-Fu-induced decrease in food intake and water intake (Fig. 2c, d). To observe the anti-senescence effect of DSV, frozen intestinal tissue sections were subjected to SA‐β‐gal staining. As indicated in the figures, three kinds of intestinal tissue sections from the 5-Fu group showed varying degrees of staining, and tissue damage could be observed. Compared to the control, 5-Fu resulted in deep staining of ileum and colonic villi and light staining of rectal villi. DSV reduced SA‐β‐gal staining in intestinal villi (Fig. 2e–g). Moreover, we evaluated the protein expression of p16 and gene expression of p21 and p53 in colon tissues. The protein expression of p16 was upregulated by 5-Fu and downregulated by DSV (Fig. 2h, i). The mRNA levels of p53 and p21 were increased in the 5-Fu group and decreased in the DSV group (Fig. 2j, k). These results showed that DSV attenuates intestinal senescence in 5-Fu-treated mice.

### DSV inhibits 5-Fu-induced senescence by inhibiting mTOR

**Figure 3:**
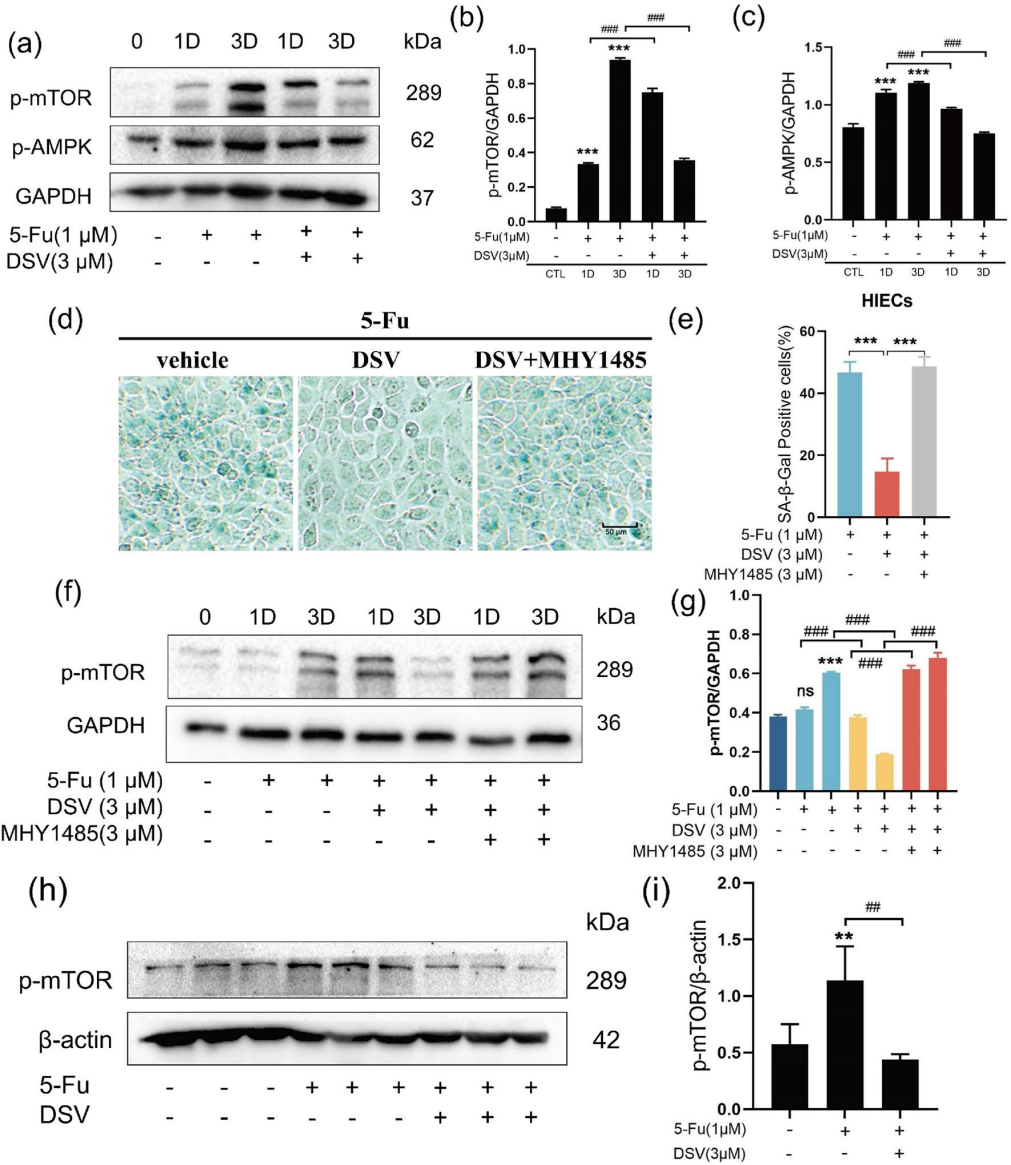
DSV inhibits 5-Fu-induced senescence by inhibiting mTOR. (**a–c**) HIECs were incubated with DSV (3 μM) and 5-Fu (1 μM) for 1 day or 3 days. Western blots and expression levels of p-mTOR and p-AMPK (n = 3). Original blots are presented in Supplementary Figs. S5, S6 and S7. (**d,e**) representative images and percentage of SA-β-gal-positive HIECs cultured with DSV (3 μM), 5-Fu (1 μM) or MHY1485 (3 μM), respectively. (magnification ×50, scale bar: 50 µm, n = 3) (**f,g**) HIECs cultured with DSV (3 μM), 5-Fu (1 μM) or MHY1485 (3 μM) for 1 day or 3 days were collected. Western blot images and expression levels of p-mTOR (n = 3). The samples were derived from the same experiment, and the blots were processed in parallel. Original blots are presented in Supplementary Figs. S8 and S9. (**h,i**) Western blot images and expression levels of p-mTOR in mouse colon tissues (n = 3). Original blots are presented in Supplementary Figs. S10 and S11. *p < 0.05, **p < 0.01, ***p < 0.001, compared with the control group; ^#^*p* < 0.05, ^##^*p* < 0.01, ^###^*p* < 0.001, compared with the indicated group.

We next investigated the mechanism by which DSV inhibits 5-Fu-induced senescence. mTOR is a pivotal regulatory molecule that is involved in regulating senescence, and inhibition of mTOR can delay senescence. As shown in the figures, mTOR protein levels in HIECs were elevated by 5-Fu treatment on Days 1 and 3 and then downregulated by DSV treatment (Fig. 3a, b). Next, we added mTOR activator MHY1485 to HIECs based on the experiments described above^23^. MHY1485 suppressed the anti-senescence effect of DSV, as indicated by an increase in the percentage of SA-β-gal-positive cells and increased mTOR protein levels (Fig. 3d–g). In colon tissue, p-mTOR protein levels were increased by 5-Fu but decreased by DSV (Fig. 3h, i). Therefore, DSV attenuates 5-Fu-induced senescence by inhibiting the mTOR signalling pathway both in vivo and in vitro. In addition, as shown in Fig. 3a, c, the p-AMPK levels were increased by 5-Fu treatment but decreased by DSV treatment. This article only describes this phenomenon without investigating the underlying mechanisms.

### DSV inhibits 5-Fu-induced abnormal levels of inflammatory factors and antioxidant enzymes in HIECs

**Figure 4:**
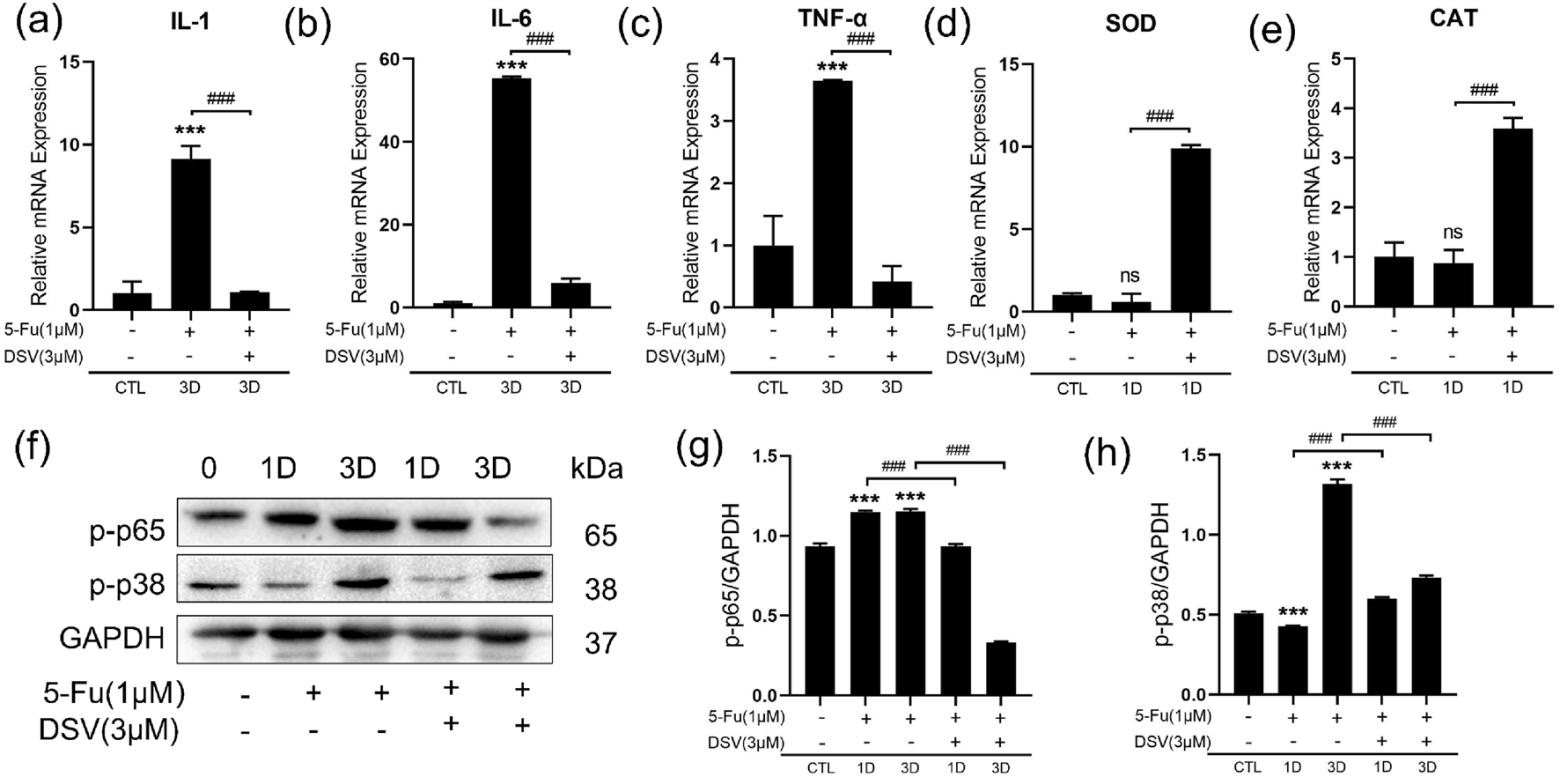
DSV inhibits 5-Fu-induced inflammatory process and oxidative stress in HIECs. (**a–e**) HIECs cultured with DSV (3 μM) and 5-Fu (1 μM) for 3 days were collected. Representative mRNA levels of IL-1β, IL-6, TNF-α, SOD and CAT were measured (n = 3). (**f–h**) HIECs cultured with DSV (3 μM) or 5-Fu (1 μM) for 1 day or 3 days were collected. Western blot images and expression levels of p-p65 and p-p38 (n = 3). The samples were derived from the same experiment, and the blots were processed in parallel. Original blots are presented in Supplementary Figs. S12, S13 and S14. *p < 0.05, **p < 0.01, ***p < 0.001, compared with the control group; ^#^*p* < 0.05, ^##^*p* < 0.01, ^###^*p* < 0.001, compared with the indicated group.

Inflammatory cytokines, such as IL-1, IL-6, and TNF-α, play key roles in the initiation and maintenance of cell senescence. Decreases in the levels of antioxidant enzymes such as Catalase (CAT) and Superoxide Dismutase (SOD) induce senescence^24^. We measured changes in the expression levels of these indicators in HIECs. After HIECs were treated with 5-Fu, the IL-1, IL-6, and TNF-α levels were increased on Day 3, and the SOD and CAT levels decreased on Day 1. However, DSV prevented these changes (Fig. 4a–e). To further investigate the anti-inflammatory effects of DSV, HIECs were treated with 1 μM 5-Fu and 3 μM DSV for 1 day and 3 days. The p-p65 and p-p38 protein levels were upregulated by 5-Fu but downregulated by DSV (Fig. 4f–h).

### DSV inhibits 5-Fu-induced intestinal injury and abnormal levels of inflammatory factors and antioxidant enzymes in mice

**Figure 5:**
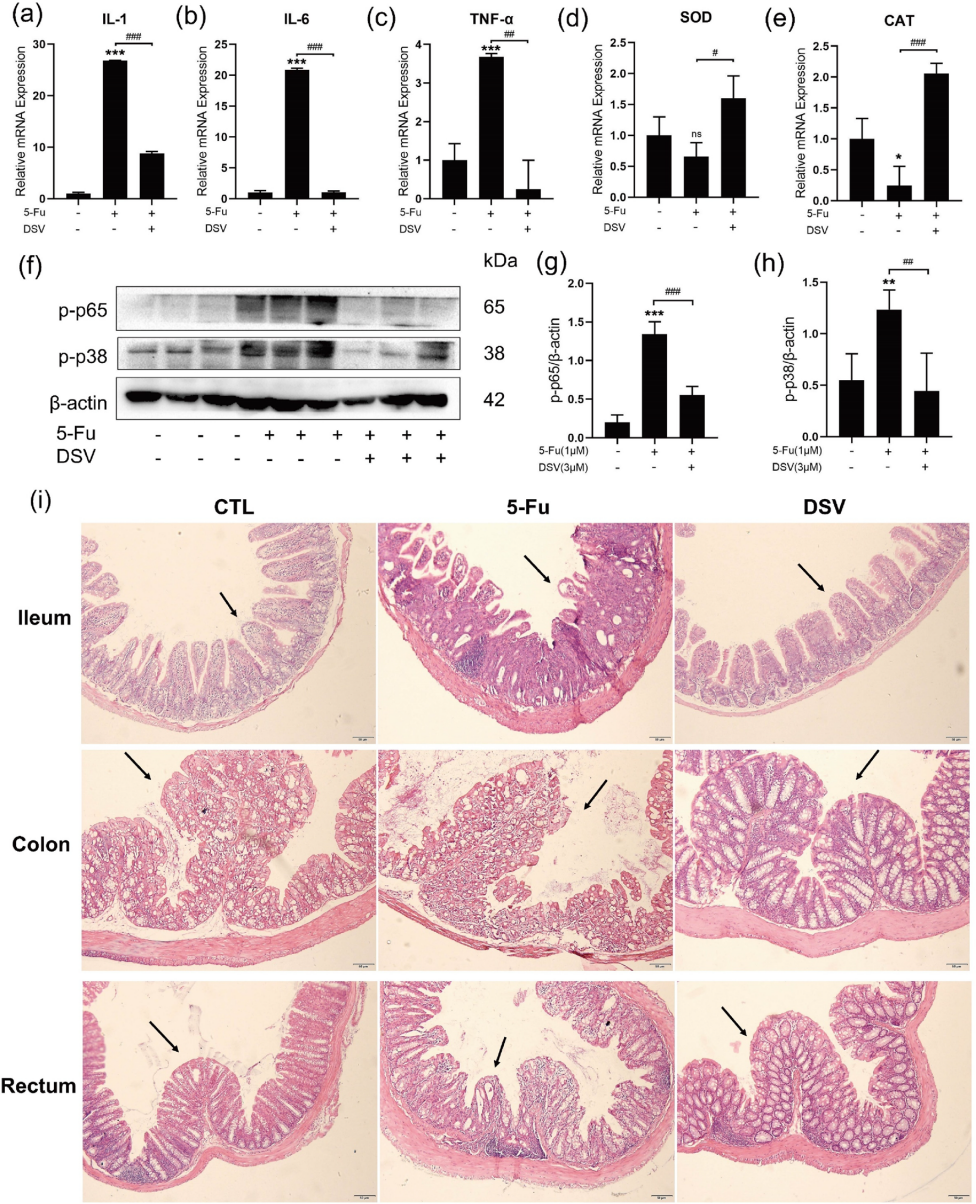
DSV inhibits 5-Fu-induced inflammatory process and oxidative stress in mice’ colon tissue. (**a–e**) Representative mRNA levels of IL-1β, IL-6, TNF-α, SOD and CAT were measured (n = 3). (f**–h**) Western blot images and expression levels of p-p65 and p-p38 (n = 3). The samples were derived from the same experiment, and the blots were processed in parallel. Original blots are presented in Supplementary Figs. S15, S16 and S17. (**i**) Haematoxylin–eosin (HE) staining of intestinal tissue sections (magnification ×100, scale bars 100 μm, n = 3) *p < 0.05, **p < 0.01, ***p < 0.001, compared with the control group; ^#^*p* < 0.05, ^##^*p* < 0.01, ^###^*p* < 0.001, compared with the indicated group.

We next examined the changes in the indicators described above in colon tissue. 5-Fu increased the levels of IL-1, IL-6, and TNF-α and decreased the levels of SOD and CAT. Treatment with DSV prevented these changes (Fig. 5a–e). 5-Fu treatment increased the p-p65 and p-p38 protein levels in mouse colon tissues, whereas DSV treatment prevented these changes (Fig. 5f–h). To observe the changes in intestinal morphology, HE staining was performed on colon, ileum and rectum samples from each group. The intestinal tissues of the control group had a normal intestinal structure, with intact villi, mucosal submucosa and basal layer. 5-Fu treatment destroyed the intestinal villi and structures, while DSV ameliorated this damage (Fig. 5i). These results demonstrated that DSV alleviates 5-Fu-induced oxidative stress, inflammation and intestinal injury induced by the above factors.

### DSV increases 5-Fu cytotoxicity in HCT116 cells

**Figure 6:**
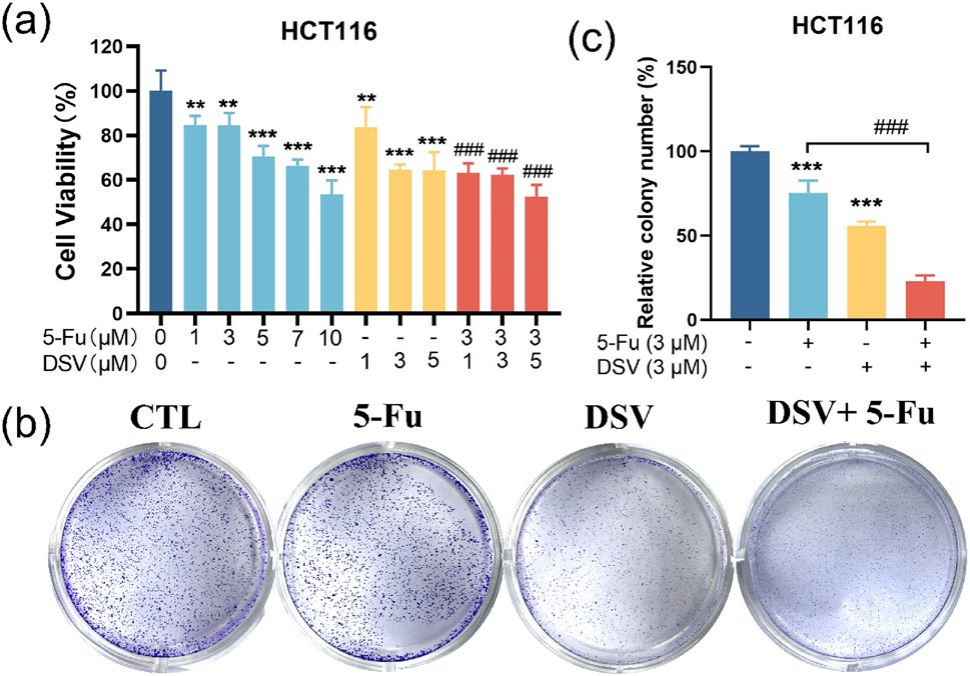
In cellular models, DSV can increase 5-Fu cytotoxicity in HCT116 cells. (**a**) HCT116 cells were treated with different concentrations of 5-Fu, DSV, or combination for 72 h. Cell viability was determined using the CCK-8 assay (n = 5). (**b,c**) HCT116 cells were treated with 3 μM 5-Fu, 3 μM DSV and combination for 72 h. The colonies were stained with crystal violet after 10 days (n = 3), and the number of colonies was quantified using ImageJ 1.52i (NIHR, USA). Finally, the relative colony number is calculated. *p < 0.05, **p < 0.01, ***p < 0.001, compared with the control group; ^#^*p* < 0.05, ^##^*p* < 0.01, ^###^*p* < 0.001, compared with 5-Fu group.

To investigate the effect of DSV on the antitumour effects of 5-Fu, we examined the synergistic effect of DSV on the anticancer properties of 5-Fu. We examined the effects of 5-Fu and DSV, alone and combined, on cell viability in HCT116s. The findings indicated that 5-Fu and DSV reduce HCT116 cell viability in a concentration-dependently, respectively. The coadministration of 5-Fu and DSV had a more pronounced cytotoxic effect on cancer cells than single-drug treatments (Fig. 6a). Subsequent experiments involved treating HCT116 cells with 3 µM 5-Fu in combination with 3 µM DSV to assess the effects on colony formation. Furthermore, colony formation assays revealed significant inhibition of cancer cell colony formation by the combined treatment (Fig. 6b, c), suggesting that the combination of 5-Fu and DSV could effectively suppress the proliferation of colorectal cancer cells.

## Discussion

An increasing number of studies have suggested that chemotherapy and radiotherapy can induce cell senescence, which leads to side effects and affects therapeutic efficacy^25^. In this study, we demonstrated that 5-Fu induced the senescence of HUVECs, HIECs and mouse intestinal tissues. Moreover, 5-Fu increased the levels of senescence-related markers. DSV is a nonstructural (NS) 5B nonnucleoside hepatitis C virus inhibitor that is used in combination with other direct antiviral agents for the treatment of chronic hepatitis C virus infection, and it has a high cure rate and few side effects^26^. Interestingly, we found that DSV alleviated 5-Fu-induced senescence in endothelial cells, intestinal epithelial cells and mouse intestinal tissues via inhibition of the mTOR signalling pathway. Moreover, DSV alleviated 5-Fu-induced inflammation and oxidative stress both in vitro and in vivo. Intestinal mucositis induced by 5-Fu in mice results in clinical manifestations including diarrhoea, weight loss, and intestinal tissue damage^27^, which were also observed in this study; these changes were ameliorated by DSV treatment. In conclusion, DSV is expected to be used as a drug for the treatment of 5-Fu-induced intestinal injury.

According to the results, 5-Fu activated mTOR in intestinal epithelial cells and mouse colon tissues. mTOR signalling contributes to the regulation of cellular senescence^28^. As an mTOR inhibitor, rapamycin slows the process of senescence and the occurrence and progression of age-related diseases in mammals^29,30^. Inhibiting mTOR affects the expression levels of inflammatory factors. Rapamycin suppresses the secretion of TNF-α, IL-1 and IL-3^31^. mTOR inhibitors have therapeutic effects on inflammatory bowel disease^32^. mTOR activation was observed in the colon of Crohn's disease patients^33^. Overactivation of mTOR leads to the apoptosis of mouse intestinal epithelial cells and induces colitis^34^. Inhibition of p-mTOR expression can treat ulcerative colitis and reduce intestinal damage. We demonstrated that DSV exerts an anti-senescence effect by inhibiting mTOR signalling, which alleviates intestinal damage.

NF-κB, which consists of several transcription factors (p52, p65, p17, c-REL and ReIB), participates in the regulation of homeostasis, inflammation and immunity^35,36^. Activation of p65 has been observed in colitis, and inhibition of NF-κB may treat inflammatory bowel disease^37^. Inhibition of NF-κB activity can reduce the 5-Fu-induced increase in the IL-1β and TNF-α levels and reduce intestinal injury^38^. The p65 pathway is also involved in the regulation of cellular senescence. Studies have shown that p65 activation is a key pathway for MSC senescence and increased inflammatory cytokine expression^39^. NF-κB is a strong signal that triggers the secretion of SASP components^40^. Senescent cells secrete many inflammatory cytokines through the p38MAPK and p65 pathways^41^. Our results demonstrated that DSV suppressed the 5-Fu-induced increase in the levels of SASP proteins (IL-1β, IL-6, and TNF-α), p-p65, and p38MAPK.

The pathogenesis of mucositis is complex and involves many processes. In addition to inflammatory processes, oxidative stress also causes apoptosis and tissue damage. Reactive oxygen species directly induce tissue damage and inflammatory processes^10,42^. Oxidative stress is also a characteristic of senescence, manifesting as increased levels of oxidative factors and decreased levels of antioxidant enzymes^24^. In our previous study, we demonstrated that 5-Fu led to an increase in ROS levels^21^. Enhancing the activity of antioxidant enzymes can alleviate oxidative stress damage^43^. The expression levels of two antioxidant enzymes (SOD and CAT) were decreased by 5-Fu but increased by DSV.

The senescence-inducing effect of chemotherapeutic drugs during cancer treatment has gradually attracted increasing attention. Inflammatory factors that are secreted by senescent cells participate in the maintenance of senescence, gradually leading to chronic inflammation. Chronic inflammation not only reduces the efficacy of chemotherapy but also causes side effects, which impose health and economic burdens on patients^25^. Some studies have shown that cells with TIS and SASP factors that are secreted by cells with TIS lead to lung inflammation, bone marrow suppression and heart failure in mice, and the removal of senescent cells can delay the progression of adverse reactions^44,45^. Our present study showed that inhibiting 5-Fu-induced intestinal senescence alleviated intestinal damage and diarrhoea. Moreover, DSV can enhance the cytotoxicity of 5-FU to HCT116 and enhance the anticancer effect.

We studied the efficacy of DSV in treating 5-Fu-induced intestinal mucositis. In conclusion, DSV alleviated 5-Fu-induced intestinal mucositis by inhibiting senescence, inhibiting inflammation, regulating oxidative stress and relieving clinical symptoms. These findings suggest that DSV can be used as an anti-senescence drug to treat 5-Fu-induced intestinal mucositis.

## Methods

### Animals

Eighteen male BALB/C mice (aged 6–8 weeks; weighing 20–25 g) were obtained from Kunming ChuShang Technology Co., Ltd. (Kunming, China). The mice were housed in individually ventilated cages with one mouse per cage. The mice were bred under standard laboratory conditions (22 ± 2 °C, 12-h light‒dark cycle). The mice were provided clean drinking water and standard chow (MD17122, Jiangsu Medicine Biopharmaceutical Co., Jiangsu, China) containing 18% protein, 53% carbohydrate, 4% fat, and 5% mineral and vitamin mixture. All the experimental protocols were approved by Dali University's Animal Protection and Ethics Committee (IACUC #2022-P2-21, Dali, Yunnan, China). All the methods were performed in accordance with the relevant guidelines and regulations.

### Induction of intestinal mucositis and treatment protocol

The animal experiments that were conducted for this study were carried out in accordance with the ARRIVE guidelines, and the details are as follows. Eighteen mice were randomly divided equally into three groups using the rand () function of Excel (Microsoft). The control group received phosphate-buffered saline (PBS, B548117, Sangon Biotech, Shanghai); the 5-Fu group received 40 mg/kg 5-Fu (H12020959, Tianjin Jinyao Pharmaceutical Co.) through intraperitoneal injection; and the DSV group received 5-Fu similar to the 5-Fu group and received 10 mg/kg DSV (S7650, Selleck, Shanghai, China). The mice in the three groups were treated by intraperitoneal injection. 5-Fu was given once every two days (on Days 2, 4, 6, 8, and 10), while DSV was given once daily. The dosage of 5-Fu that was used in this study was based on our published article^21^. The dosage of DSV was selected according to a published article and our preliminary experiment^46^. For each animal, three different investigators were involved: two investigators (S.H. and J.X.) administered the treatment based on the randomization table, and a third investigator (D.Q.) recorded changes in body weight, food intake and water intake at the same time every day. The stool of the mice was evaluated according to the following criteria, and the diarrhoea score was recorded by an investigator who was blinded to the treatments. The diarrhoea score was used to assess the severity of diarrhoea based on the following criteria: 0: normal stool; 1: squishy stool (mild diarrhoea); 2: moist and unshaped stool (moderate diarrhoea); and 3: liquid stool (severe diarrhoea)^43^. After the experiment, the mice were sacrificed by cervical dislocation of the neck, and the colon, ileum, and rectal tissues were collected from each mouse. The intestinal tissues of three mice in each group were used to generate frozen sections and paraffin sections, and the intestinal tissues of the other three mice were used for immunoblotting and RT‒qPCR experiments. Therefore, each intestinal tissue sample was considered an experimental unit. No exclusion or inclusion criteria were applied to the mice that were used in this study.

### Cell drug treatments

Dasabuvir was first dissolved in dimethyl sulfoxide (DMSO) at 1000 mM (stock concentration) and then diluted with culture medium to the appropriate concentration. The final concentration of DMSO was less than 0.1%.

### Cell culture

Human intestinal epithelial cells (HIECs, Guangzhou Jennio Biotech Co. L.T.D.), human colorectal cancer cells (HCT116, Kunming Cell Bank, Chinese Academy of Sciences, Kunming, China) were cultured in DMEM supplemented with 10% foetal serum, 100 units/ml penicillin plus streptomycin and 2 mM l-glutamine. Human umbilical vein endothelial cells (HUVECs, Chinese Academy of Sciences Biological Sciences, Shanghai, China) were cultured in RPMI-1640 supplemented with 10% foetal serum, 100 units/ml penicillin plus streptomycin and 2 mM l-glutamine. The cells were cultured in a humid environment (37 °C, 5% CO_2_).

### Cell viability assay

HUVECs, HIECs, and HCT116 cells were separately cultured in 96-well plates (1000 cells per well, 3 replicate wells, 24 h), and each group was cultured with the indicated drugs for 72 h. After incubation, the medium was removed, and a mixture of CCK-8 and medium (1:9 volume ratio) was added to each well at 100 μL/well and incubated for 1 h. A microplate absorbance reader (Bio-Rad, USA) was used to measure the optical density (OD) at 450 nm. The concentrations of the above reagents are indicated in the corresponding figure legends.

### Colony-formation efficiency assay

HCT116 cells were seeded in six-well plates at a density of 2000 cells per well and cultured with the indicated concentrations of DSV, 5-Fu or the combination for 72 h. After incubation, the cells were cultured in drug-free medium for 10 days. After colonies formed, the cells were fixed with 4% paraformaldehyde for 15 min and stained with 0.1% crystal violet for 15 min. The colonies were counted and analyzed using ImageJ 1.52i (NIHR, USA).

### SA-β-Gal staining

After the mice were sacrificed, intestinal tissues were fixed in OCT at low temperature and sliced into 4-μm sections. The SA-beta-gal activity of HUVECs, HIECs and intestinal samples was measured using a senescence-associated-β-galactosidase (SA-β-Gal) staining kit according to the manufacturer's instructions. The senescent cells and sections were identified by blue/green staining under an optical microscope, and images were captured. The percentage of SA-β-Gal-positive cells was determined by counting 1,000 cells in 7 random fields for each group.

### Histological staining

Intestinal tissues were fixed in 4% paraformaldehyde and embedded in paraffin blocks. Paraffin-embedded tissue sections (5 µm thick) were deparaffinized and stained using a haematoxylin–eosin (HE) staining kit (Solarbio, Beijing, China). The samples were viewed and images were acquired using a light microscope.

### Immunoblotting

Collected HIEC cells and colon samples were lysed using a premixture of RIPA lysis buffer (G2002, Solarbio, China) and phenyl methane sulfonyl fluoride (PMSF, G2008, Solarbio, China) at a ratio of 100:1. Protein concentrations were determined by the BCA method. Protein samples were separated on SDS‒PAGE gels and transferred to PVDF membranes. After blocking with 5% skim milk for 1 h, the membranes were incubated with primary antibodies (1:1000; p-mTOR, 67778-1-Ig; p16, 10883-1-AP; p-p38, 28796-1-AP; p-p65, KHC0634; and p-AMPK, 10929-2-AP; Proteintech, Wuhan, Hubei) overnight. After incubation with the corresponding secondary antibody (1:10,000, SA00001-2, Proteintech, Wuhan, Hubei), the bands were visualized by a ChemiDoc imaging system and quantified by ImageJ 1.52i (NIHR, USA).

### Real-time-quantitative polymerase chain reaction (RT-qPCR)

After the indicated treatments, HIECs and colon samples were lysed with TRIzol reagent (R0016, Beyotime, China) to isolate total RNA. Total RNA (2 g) was used to synthesize cDNA using a reverse transcriptase kit (R333, Vazyme, China). The synthesized cDNA was amplified by qRT-PCR with SYBR Green qPCR Master Mix (2×, HY-K0521, China) on a StepOne Real-Time PCR System (Applied Biosystems, USA). 18S gene expression was used as a housekeeping gene. The primer sequences are shown in Table 1 at the end of the manuscript.

**Table 1:** Primer sequences.

Target gene	Primer sequence (5'–3')
h-IL-1β	F: TTCGACACATGGGATAACGAGG R: TTTTTGCTGTGAGTCCCGGAG
h-IL-6	F: CCTGAACCTTCCAAAGATGGC R: TTCACCAGGCAAGTCTCCTCA
h-TNF-α	F: GAGGCCAAGCCCTGGTATG R: CGGGCCGATTGATCTCAGC
h-p53	F: ACAGCTTTGAGGTGCGTGTTT R: CCCTTTCTTGCGGAGATTCTCT
h-p21	F: CGATGGAACTTCGACTTTGTCA R: GCACAAGGGTACAAGACAGTG
h-CAT	F: TGGGATCTCGTTGGAAATAACAC R: TCAGGACGTAGGCTCCAGAAG
h-SOD	F: TTTCAATAAGGAACGGGGACAC R: GTGCTCCCACACATCAATCC
m-IL-1β	F: CTGTGACTCATGGGATGATGATG R: CGGAGCCTGTAGTGCAGTTG
m-IL-6	F: TAGTCCTTCCTACCCCAATTTCC R: TTGGTCCTTAGCCACTCCTTC
m-TNF-α	F: CCTGTAGCCCACGTCGTAG R: GGGAGTAGACAAGGTACAACCC
m-p53	F: GGCAGACTTTTCGCCACAG R: CAGGCACAAACACGAACCTC
m-p21	F: CGCTGTCTTGCACTCTGGT R: CGTTTTCGGCCCTGAGATGTT
m-CAT	F: AGCGACCAGATGAAGCAGTG R: TCCGCTCTCTGTCAAAGTGTG
m-SOD	F: CCAAGGGAGATGTTACAACTCAG R: GGGCTCAGGTTTGTCCAGAA

### Statistical analysis

All the data are presented as the mean ± SD of at least three biological replicates. Sample size for each experiment is described in the corresponding figure legend. Differences between groups were compared using Student’s t test. Several group comparisons were performed using one-way ANOVA with the Bonferroni post hoc correction. Statistics of Fig. 2a–d were determined by Two-way ANOVA. All the statistical analyses were performed using GraphPad Prism 8.0.2 (GraphPad Sofware, Inc., San Diego, CA, USA), and p < 0.05 was considered to indicate statistical significance.

### Supplementary Information

Supplementary Figures.

## Data Availability

The data that support the findings of this study are available from the corresponding author upon reasonable request.

## Abbreviations

AMPK: Adenosine 5′monophosphate-activated protein kinase
DSV: Dasabuvir
NF-kappa B: Nuclear factor-kappa B
p38MAPK: P38 mitogen-activated protein kinase
TNF-α: Tumor necrosis factor-alpha
CTL: Control
DMEM: Dulbecco's modified Eagle's medium
RPMI1640: Roswell Park Memorial Institute 1640
DMSO: Dimethyl sulfoxide
mTOR: Mechanistic target of rapamycin
ROS: Reactive oxygen species
SASP: Senescence-associated secretory phenotype
HIEC: Human intestinal epithelial cell
HUVEC: Human umbilical vein endothelial cell
HCT116: Human colorectal cancer cell
