# Supplementary material for: Dasabuvir alleviates 5-fluorouracil-induced intestinal injury through anti-senescence and anti-inflammatory
Source: Sci Rep. 2024 Jul 8;14:15730. doi: 10.1038/s41598-024-66771-x (PMC11231161; doi:10.1038/s41598-024-66771-x)

Dasabuvir alleviates 5-Fluorouracil-induced intestinal injury through anti-senescence and anti-inflammatory

Siyue He, Zhiwei Wang, Jing Xia, Huijie-Jia, Qianlong Dai, Cui Chen, Fei He, Xiaobo Wang, Min Zhou

Fig.1. DSV inhibits senescence induced by 5-FU in HUVECs and HIECs.

p16 and GAPDH protein expression levels in HIEC cells of four groups (control group, 5-Fu group, DSV group and 5-Fu+DSV group, Day1 and Day3). The full-length membrane was cut out of the region containing the band of interest with reference to the markers used as indicators that were simultaneously appliqued during electrophoresis. The samples derive from the same experiment and that blots were processed in parallel. The edges of the membrane have been indicated by arrows.

Supplementary Figure S1: Fig 1j - p16 (16 kDa) HIEC cell sample

0 1D 3D 1D 3D 1D 3D

15kDa

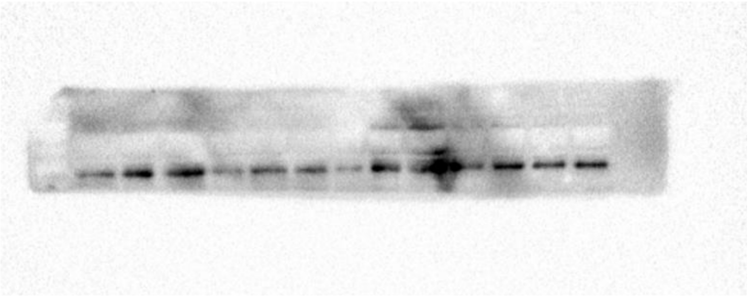

HIEC cell sample

p16:16 kDa

5-Fu (1 μM) - + + - - + +

DSV (3 μM) - - - + + + +

Supplementary Figure S2: Fig 1j - GAPDH (37 kDa) HIEC cell sample

0 1D 3D 1D 3D 1D 3D

35kDa

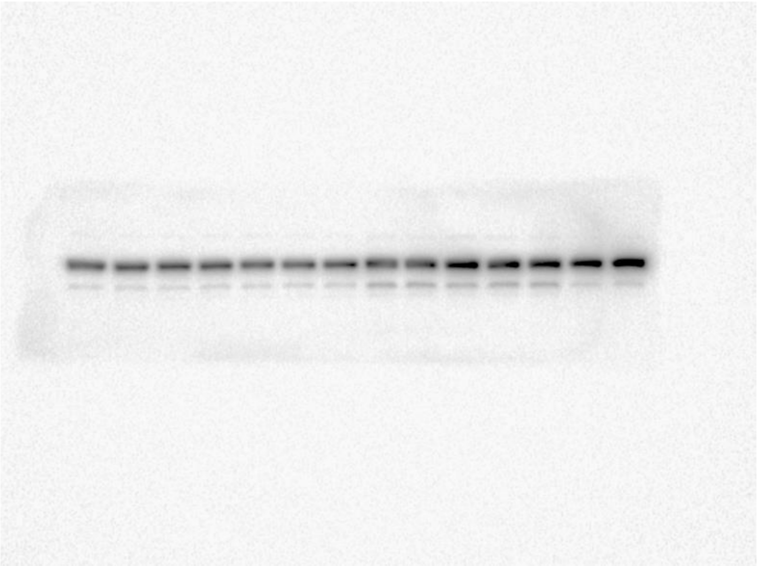

HIEC cell sample

GAPDH:37 kDa

5-Fu (1 μM) - + + - - + +

DSV (3 μM) - - - + + + +

Fig.2. DSV inhibits intestinal senescence induced by 5-FU.

p16 and β-actin protein expression levels in colon tissue of three groups (control group, 5-Fu group, and 5-Fu+DSV group). The full-length membrane was cut out of the region containing the band of interest with reference to the markers used as indicators that were simultaneously appliqued during electrophoresis. The samples derive from the same experiment and that blots were processed in parallel. The edges of the membrane have been indicated by arrows.

Supplementary Figure S3: Fig 2h – p16 (16 kDa) colon tissue sample

15kDa

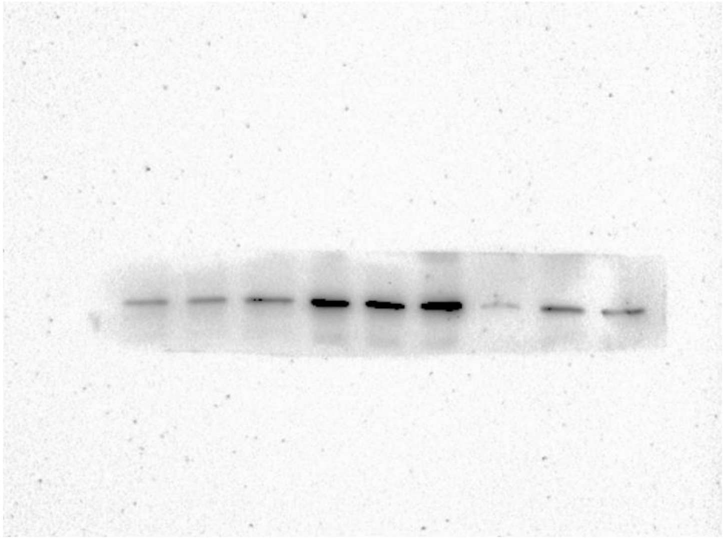

colon tissue sample

p16:16 kDa

5-Fu	-	-	-	+	+	+	+	+	+
DSV	-	-	-	-	-	-	+	+	+

Supplementary Figure S4: Fig 2h – β-actin (42 kDa) colon tissue sample

40kDa

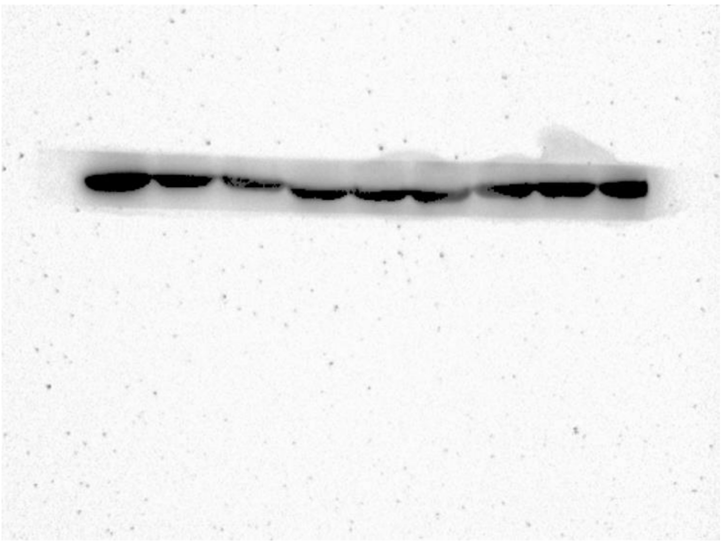

colon tissue sample

β-actin: 42 kDa

5-Fu	-	-	-	+	+	+	+	+	+
DSV	-	-	-	-	-	-	+	+	+

Fig.3. DSV inhibits 5-FU-induced senescence by inhibiting mTOR

p-mTOR, p-AMPK and GAPDH protein expression levels in HIEC cells of three groups (control group, 5-Fu group, and 5-Fu+DSV group, Day1 and Day3). The full-length membrane was cut out of the region containing the band of interest with reference to the markers used as indicators that were simultaneously appliqued during electrophoresis. The samples derive from the same experiment and that blots were processed in parallel. The edges of the membrane have been indicated by arrows.

Supplementary Figure S5: Fig 3a – p-mTOR (289 kDa) HIEC cells sample

0 1D 3D 1D 3D

180kDa

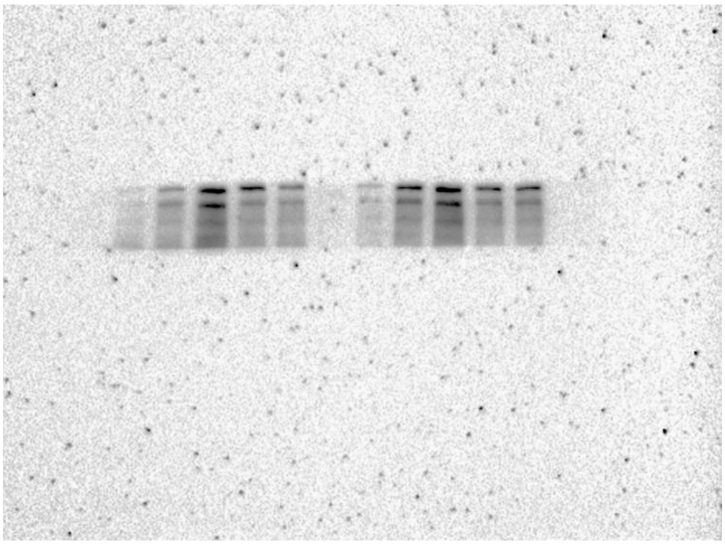

HIEC cells sample

p-mTOR:289 kDa

5-Fu (1 μM) - + + + +

DSV(3μM) - - - + +

Supplementary Figure S6: Fig 3a – p-AMPK (62 kDa) HIEC cells sample

CTL 1D 3D 1D 3D

70kDa

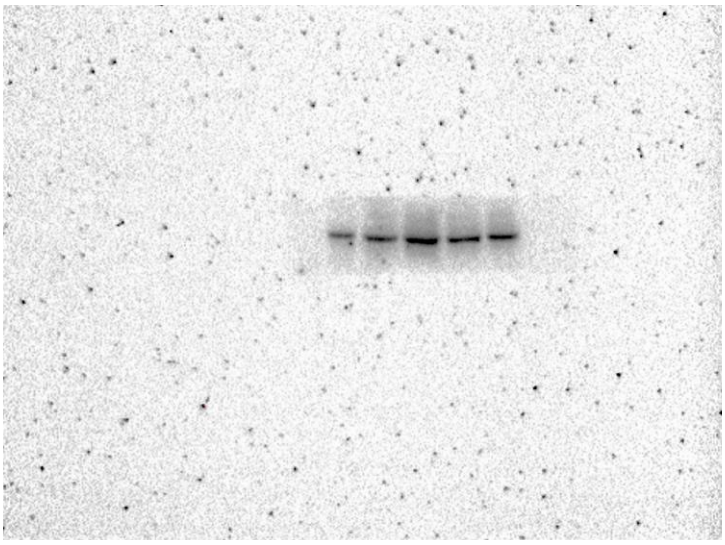

HIEC cells sample

p-AMPK :62 kDa

5-Fu (1 μM) - + + + +

DSV(3μM) - - - + +

Supplementary Figure S7: Fig 3a – GAPDH (37 kDa) HIEC cells sample

CTL 1D 3D 1D 3D

35kDa

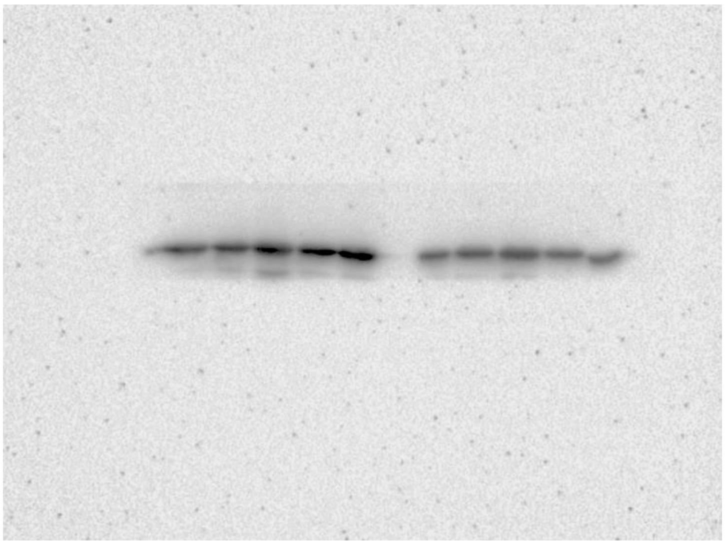

HIEC cells sample

GAPDH:37 kDa

5-Fu (1 μM)	-	+	+	+	+
DSV(3μM)	-	-	-	+	+

p-mTOR and GAPDH protein expression levels in HIEC cells of four groups (control group, 5-Fu group, 5-Fu+DSV group and 5-Fu+DSV+MHY1485 group, Day1 and Day3). The full-length membrane was cut out of the region containing the band of interest with reference to the markers used as indicators that were simultaneously appliqued during electrophoresis. The samples derive from the same experiment and that blots were processed in parallel. The edges of the membrane have been indicated by arrows.

Supplementary Figure S8: Fig 3f – p-mTOR (289 kDa) HIEC cells sample

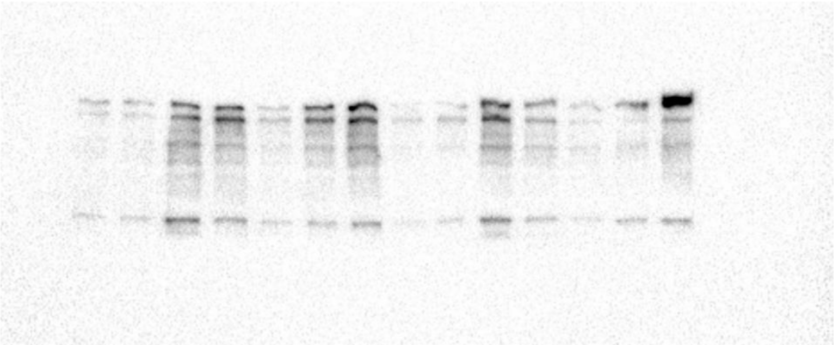

Supplementary Figure S9: Fig 3f – GAPDH (37 kDa) HIEC cells sample

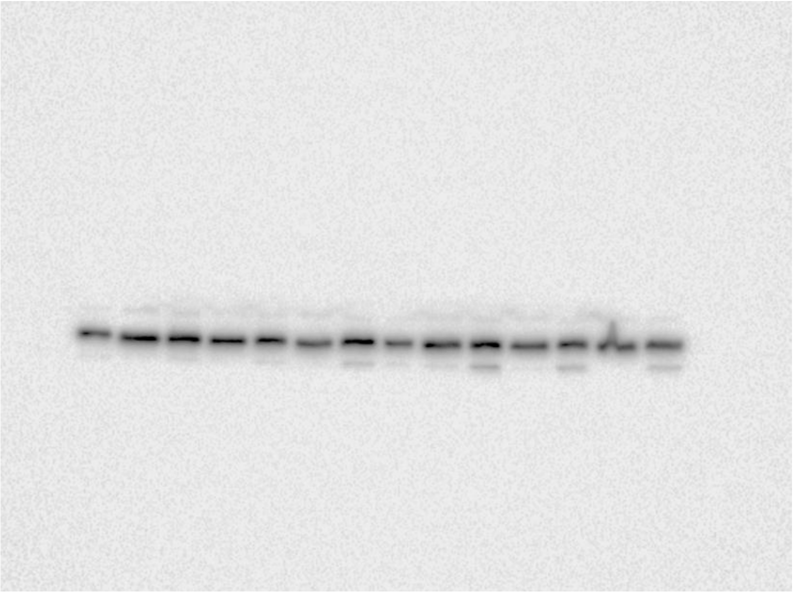

p-mTOR and β-actin protein expression levels in colon tissue of three groups (control group, 5-Fu group, and 5-Fu+DSV group). The full-length membrane was cut out of the region containing the band of interest with reference to the markers used as indicators that were simultaneously appliqued during electrophoresis. The samples derive from the same experiment and that blots were processed in parallel. The edges of the membrane have been indicated by arrows.

Supplementary Figure S10: Fig 3h – p-mTOR (289 kDa) colon tissue sample

180kDa

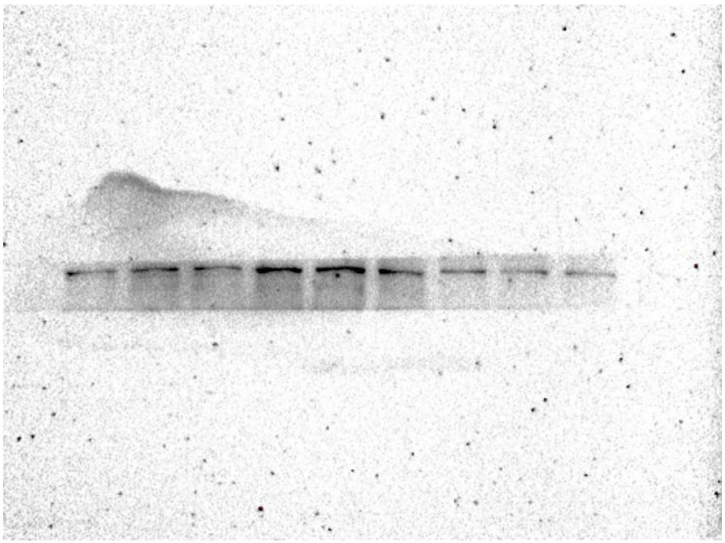

colon tissue sample
p-mTOR: 289 kDa

5-Fu	-	-	-	+	+	+	+	+	+
DSV	-	-	-	-	-	-	+	+	+

Supplementary Figure S11: Fig 3h – β-actin (42 kDa) colon tissue

40kDa

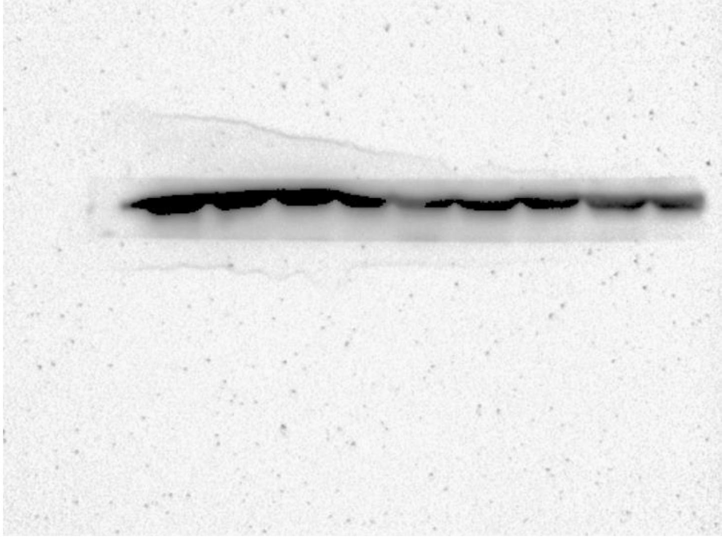

colon tissue sample
β-actin:42 kDa

5-Fu	-	-	-	+	+	+	+	+	+
DSV	-	-	-	-	-	-	+	+	+

Fig.4. DSV inhibits 5-FU-induced inflammatory process and oxidative stress in HIECs.

p-p65, p-p38 and GAPDH protein expression levels in HIEC cells of three groups (control group, 5-Fu group, and 5-Fu+DSV group, Day1 and Day3). The full-length membrane was cut out of the region containing the band of interest with reference to the markers used as indicators that were simultaneously appliqued during electrophoresis. The samples derive from the same experiment and that blots were processed in parallel. The edges of the membrane have been indicated by arrows.

Supplementary Figure S12: Fig 4f – p-p65 (65 kDa) HIEC cells sample

CTL 1D 3D 1D 3D

70kDa

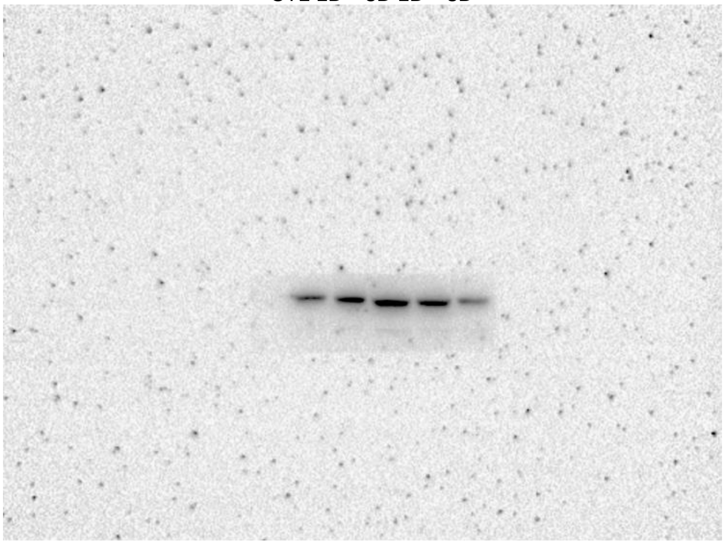

HIEC cells sample
p-p65:65 kDa

5-Fu(1μM) - + + + +
DSV(3μM) - - - + +

Supplementary Figure S13: Fig 4f – p-p38 (38 kDa) HIEC cells sample

CTL 1D 3D 1D 3D

40kDa

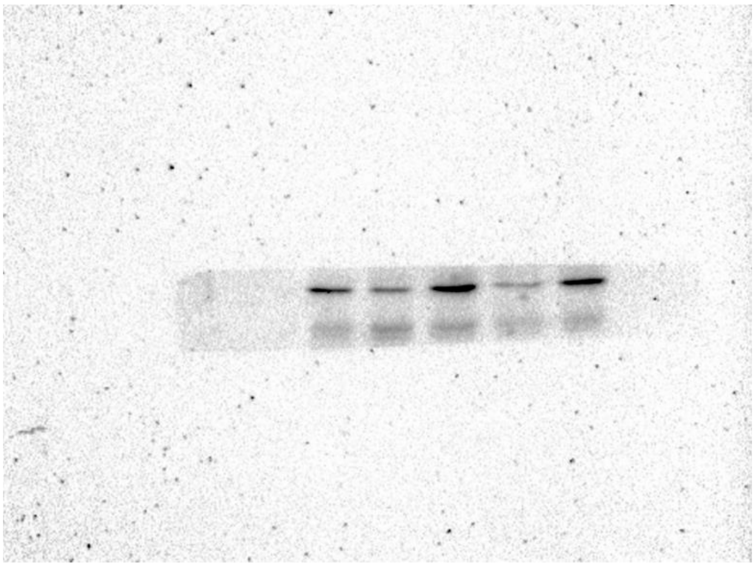

HIEC cells sample
p-p38:38 kDa

5-Fu(1μM) - + + + +
DSV(3μM) - - - + +

Supplementary Figure S14: Fig 4f – GAPDH (37 kDa) HIEC cells sample

CTL 1D 3D 1D 3D

35kDa

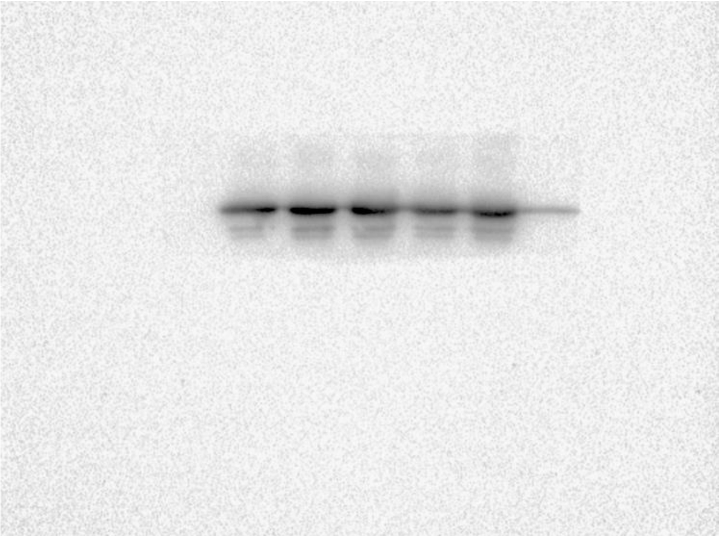

HIEC cells sample

GAPDH:37 kDa

5-Fu(1μM)	-	+	+	+	+
DSV(3μM)	-	-	-	+	+

Fig. 5. DSV inhibits 5-FU-induced inflammatory process and oxidative stress in mice' colon tissue.

p-p65, p-p38 and β-actin protein expression levels in colon tissue of three groups (control group, 5-Fu group, and 5-Fu+DSV group). The full-length membrane was cut out of the region containing the band of interest with reference to the markers used as indicators that were simultaneously appliqued during electrophoresis. The samples derive from the same experiment and that blots were processed in parallel. The edges of the membrane have been indicated by arrows.

Supplementary Figure S15: Fig 5f – p-p65 (65 kDa) colon tissue sample

70kDa

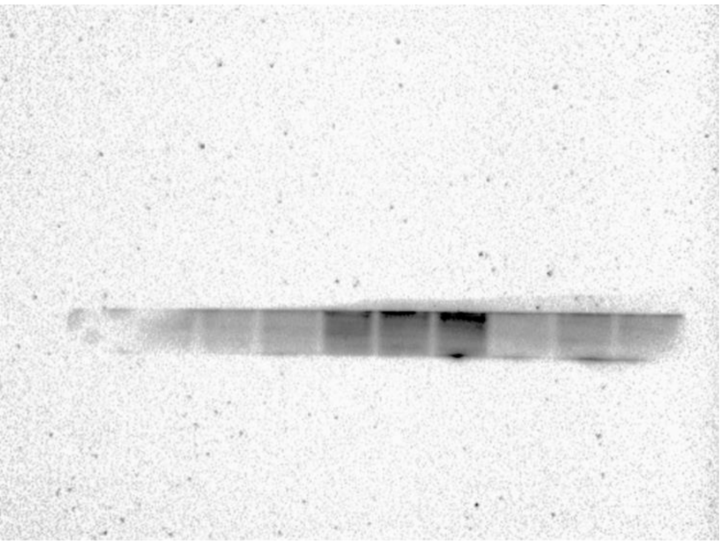

colon tissue
p-p65:65 kDa

5-Fu	-	-	-	+	+	+	+	+	+
DSV	-	-	-	-	-	-	+	+	+

Supplementary Figure S16: Fig 5f – p-p38 (38 kDa) colon tissue

35kDa

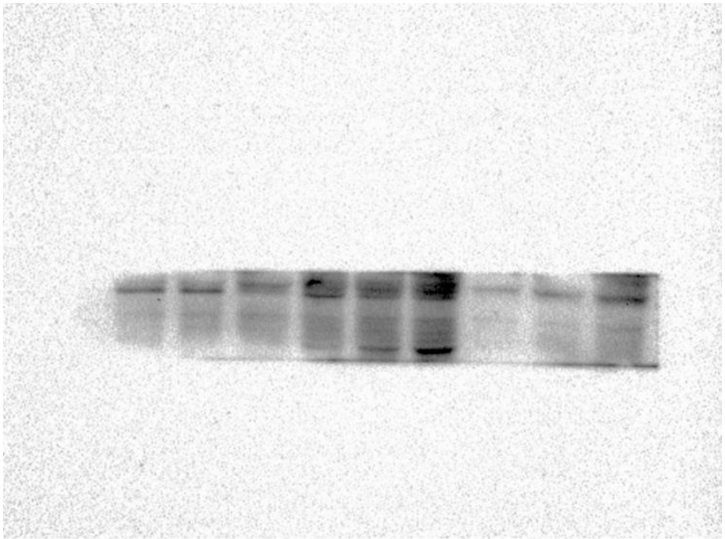

colon tissue
p-p38:38 kDa

5-Fu	-	-	-	+	+	+	+	+	+
DSV	-	-	-	-	-	-	+	+	+

Supplementary Figure S17: Fig 5f – β-actin (42 kDa) colon tissue

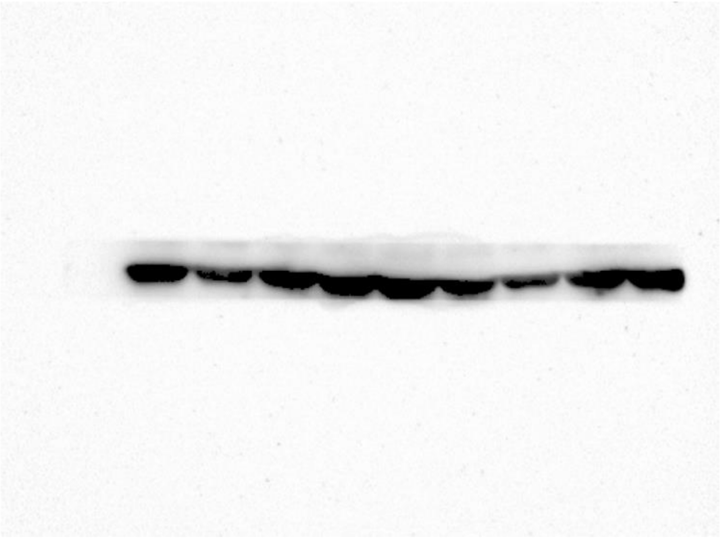

Supplement: Supplementary file 1 — Supplementary Figures. [file 41598_2024_66771_MOESM1_ESM.pdf]
